# Scaling and sustaining COVID-19 vaccination through meaningful community engagement and care coordination for underserved communities: hybrid type 3 effectiveness-implementation sequential multiple assignment randomized trial

**DOI:** 10.1186/s13012-023-01283-2

**Published:** 2023-07-14

**Authors:** Borsika A. Rabin, Kelli L. Cain, Paul Watson, William Oswald, Louise C. Laurent, Audra R. Meadows, Marva Seifert, Fatima A. Munoz, Linda Salgin, Jeannette Aldous, Edgar A. Diaz, Miguel Villodas, Santosh Vijaykumar, Sean T. O’Leary, Nicole A. Stadnick

**Affiliations:** 1 Herbert Wertheim School of Public Health and Human Longevity Science, University of California San Diego, La Jolla, CA, USA; 2 Dissemination and Implementation Science Center, University of California San Diego Altman Clinical and Translational Research Institute, La Jolla, CA, USA; 3 The Global Action Research Center, San Diego, CA USA; 4 Department of Obstetrics, Gynecology, and Reproductive Sciences, University of California San Diego, La Jolla, CA USA; 5 Department of Medicine, University of California San Diego, La Jolla, CA USA; 6 San Ysidro Health, San Diego, CA USA; 7 Department of Psychology, San Diego State University, CA San Diego, USA; 8 Child and Adolescent Services Research Center, San Diego, CA USA; 9 Department of Psychology, Northumbria University, Newcastle Upon Tyne, UK; 10 Department of Pediatrics-Infectious Diseases, University of Colorado Anschutz Medical Campus, Aurora, CO USA; 11 Department of Psychiatry, University of California San Diego, La Jolla, CA USA

**Keywords:** Implementation science, Hybrid type 3 effectiveness-implementation study, Sequential multiple assignment randomized trial, COVID-19, Underserved communities, Health equity, Care coordination, mHealth, Vaccination

## Abstract

**Background:**

COVID-19 inequities are abundant in low-income communities of color. Addressing COVID-19 vaccine hesitancy to promote equitable and sustained vaccination for underserved communities requires a multi-level, scalable, and sustainable approach. It is also essential that efforts acknowledge the broader healthcare needs of these communities including engagement in preventive services.

**Methods:**

This is a hybrid type 3 effectiveness-implementation study that will include a multi-level, longitudinal, mixed-methods data collection approach designed to assess the sustained impact of a co-created multicomponent strategy relying on bidirectional learning, shared decision-making, and expertise by all team members. The study capitalizes on a combination of implementation strategies including mHealth outreach with culturally appropriate messaging, care coordination to increase engagement in high priority preventive services, and the co-design of these strategies using community advisory boards led by Community Weavers. Community Weavers are individuals with lived experience as members of an underserved community serving as cultural brokers between communities, public health systems, and researchers to co-create community-driven, culturally sensitive public health solutions. The study will use an adaptive implementation approach operationalized in a sequential multiple assignment randomized trial design of 300 participants from three sites in a Federally Qualified Health Center in Southern California. This design will allow examining the impact of various implementation strategy components and deliver more intensive support to those who benefit from it most. The primary effectiveness outcomes are COVID-19 vaccine completion, engagement in preventive services, and vaccine confidence. The primary implementation outcomes are reach, adoption, implementation, and maintenance of the multicomponent strategy over a 12-month follow-up period. Mixed-effects logistic regression models will be used to examine program impacts and will be triangulated with qualitative data from participants and implementers.

**Discussion:**

This study capitalizes on community engagement, implementation science, health equity and communication, infectious disease, and public health perspectives to co-create a multicomponent strategy to promote the uptake of COVID-19 vaccination and preventive services for underserved communities in San Diego. The study design emphasizes broad engagement of our community and clinic partners leading to culturally sensitive and acceptable strategies to produce lasting and sustainable increases in vaccine equity and preventive services engagement.

**Trial registration:**

ClinicalTrials.gov Identifier: NCT05841810 May 3, 2023

Contributions to the literature

- This study applies a sequential multiple assignment randomized trial design to understand and accelerate the impact of a co-created health prevention program designed to address COVID-19 vaccine equity and primary care engagement for communities of color served in a Federally Qualified Health Center.

- This study integrates methods from community engagement, implementation science, health equity, health communication, infectious disease, and public health perspectives to mitigate health disparities and advance health equity.
- This study focuses on the rarely examined implementation outcome of sustained engagement in preventive services among historically underserved communities.

## Background

### COVID-19 disparities experienced by underserved communities in California

The COVID-19 pandemic has punctuated pervasive health disparities among immigrant, refugee, Latino, Asian, and Black, Indigenous, People of Color (BIPOC) communities [1, 2]. These communities are significantly more likely to experience mortality and morbidity from COVID-19, along with lagging testing and vaccination rates compared to predominantly white communities [3]. Despite the availability of multiple safe and effective vaccines that have been recommended by the Centers for Disease Control and Prevention (CDC), vaccination rates have been slower and variable in BIPOC communities [4, 5].

Although current data on vaccination rates in the United States of America (USA) offer hope towards ending the COVID-19 pandemic (as of this writing), 85% of adults completed the primary vaccine series; 65% of eligible adults have received a booster dose in addition to completing the primary series; and vaccine uptake has lagged across BIPOC communities in San Diego County with only 51% of Black, 69% of Hispanic, and 71% of Asian residents having completed the primary vaccines series [6]. To address persistent inequities, the CDC National Forum on COVID-19 vaccine emphasizes that “barriers to vaccine access must be removed and evidence-based approaches to improving vaccine confidence and acceptance are essential” [7, 8].

### Multi-level factors driving vaccine hesitancy and vaccine uptake: individual, structural, systemic, and technological

“Vaccine uptake” is defined as the completion of a vaccination series per administration schedule as recommended by medical experts and appropriate institutions. Vaccine uptake is complex and driven by individual-level factors, structural and systemic factors, and technological factors; these impacts are amplified for underserved communities. Although vaccine uptake has increased, underserved communities have been historically overlooked by public health strategies tailored to resource-rich communities with predominantly English-speaking residents [9].

At the individual level, the research and popular literature have increasingly focused on the concept of “vaccine hesitancy.” Vaccine hesitancy refers to delay in acceptance or refusal of vaccination despite availability of vaccination services [10]. Vaccine hesitancy is the midpoint in decision-making between vaccine refusal and acceptance and can be influenced by societal/community norms, individual attitudes (e.g., complacency, confidence), and structural factors (e.g., convenience, access) [11]. A related term, “vaccine confidence,” is defined as the belief that vaccines work, are safe, and are part of a trustworthy medical system [12]. Factors driving vaccine hesitancy and confidence include medical distrust shaped by historical trauma rooted in biomedical research and continues to be reinforced by current experiences of discrimination within medical encounters. The COVID-19 pandemic has also prompted a deluge of information, opinion, and conspiracies known as the “infodemic” which has played a significant role in vaccine hesitancy [13]. Rapid and widespread sharing of both evidence-based and inaccurate information about COVID-19 and vaccines leads some people to inaction. This has been particularly salient in Spanish-language social media [14–17]. Vaccine hesitancy or confidence at the individual level is one, but often not the most, important explanatory variable of vaccine uptake [13]. Additional factors driving uptake are largely related to access, including structural and systemic barriers.

The pandemic has created an overall reduction in timeliness of immunizations and preventive service completion [18, 19] and underscored an urgent need to overcome structural barriers preventing certain populations from accessing high quality, convenient, and affordable healthcare. “Preventive services” are defined as routine, guideline-concordant health care that includes chronic health screenings, check-ups, and patient counseling to prevent illnesses, disease, or other health problems [20]. Vaccine distribution is traditionally embedded within existing healthcare infrastructure that is inequitably designed and inadequately funded. Even among those with a healthcare provider, studies show that BIPOC tend to receive lower-quality healthcare than white people after controlling for insurance status, income, age, and condition severity [21]. As a vaccine comparison, influenza vaccination rates are consistently lower among Black, Latino, and Asian American persons, as well as individuals of lower socioeconomic status (SES), compared with their white and higher SES counterparts [22]. San Diego residents that are particularly impacted by barriers to healthcare access include undocumented residents, isolated community members, individuals whose preferred language is not English, or those who need transportation and access accommodations.

Systemic and logistical factors also impact access to the COVID-19 vaccine. The digital divide (i.e., differential access to technology needed to schedule appointments and obtain updated health information) precludes a sizeable portion of the population from accessing web-based scheduling portals to set up vaccination appointments. Individuals also need to access information in their preferred languages to make informed decisions about vaccination and to schedule vaccination appointments. Compounding these structural factors, concerns about the need to take sick or unpaid leave, or secure childcare, due to vaccine side effects might negatively impact vaccine uptake.

### Promise of implementation science in addressing vaccine hesitancy and uptake for underserved communities

Addressing COVID-19 vaccine hesitancy to promote equitable and sustained vaccination for underserved communities requires a multi-level, scalable, and sustainable approach. It is also essential that efforts acknowledge the broader healthcare needs of underserved communities including engagement in preventive services [23–25]. The current study builds on the project team’s community-engaged infrastructure to conduct a hybrid type 3 effectiveness-implementation trial [26, 27] to examine the impact of a co-created multicomponent implementation strategy on increased and sustained COVID-19 vaccination and engagement in preventive services for underserved communities within a FQHC network. This work will combine methods from implementation science, health equity research, and community engagement.

### Implementation strategies

This study is a hybrid type 3 effectiveness-implementation trial in which the evidence-based interventions are COVID-19 vaccines and age- and gender-specific recommended preventive care services. The three implementation strategies that support the uptake of these evidence-based interventions are as follows: (1) co-creation with community advisory boards (CABs) led by Community Weavers, (2) the use of mobile health (mHealth) technologies for health communication, and (3) care coordination. The next section provides a brief overview of each implementation strategy.

#### Co-creation with CABs led by Community Weavers

Meaningful community engagement is an important factor in facilitating the uptake, implementation, and sustained use of interventions designed to address health inequities [28, 29]. Community engagement, however, is not a trivial task, especially when working with communities representing diverse cultural heritages and languages and whose voices have historically been silenced [30, 31]. In the creation of community-vetted solutions to inequitable vaccine distribution and uptake, as well as access to preventive services, it is critical to utilize approaches that acknowledge and address these historical patterns [32]. *Community Weavers are individuals with lived experience as a member of an underserved community who serve as cultural brokers between communities, public health systems, and researchers to co-create community-driven, culturally sensitive public health solutions* [33, 34]. Community Weavers can bridge the gap between the underserved community and the mainstream public health systems and support research and clinical partners in learning about the priorities, preferences, needs, and strengths of the community, which can lead to solutions that are culturally sensitive and responsive [34]. They can also support the engagement of community members in health care, including COVID-19 vaccination and other preventive services. The Global Action Research Center (ARC), a nonprofit social change organization with expertise conducting participatory action research to address public health needs and a trusted resource for many of the grassroots organizations, will be the key lead in identifying, training, and engaging our Community Weavers in this project. A key component in this process is to engage in co-creation of solutions with members of the community. *Co-creation*builds on the concepts of bidirectional learning and shared decision-making and relies on the expertise brought by all members of the team [35, 36]. Co-creation has been successfully used in diverse contexts, and programs that incorporate co-creation tend to have better uptake, more comprehensive implementation, and lead to more sustained positive outcomes [37–39]. Co-creation has been at the heart of our prior community-engaged COVID-19 research with our community and clinical partners to meaningfully engage diverse community members in participatory methods.

#### Use of mobile health (mHealth) technologies for health communication

Lessons addressing challenges with vaccine distribution and vaccine hesitancy can be drawn from decades of vaccine campaign research from low- or middle-income countries (LMICs) through reverse innovation. Reverse innovation involves replicating an innovation that was originally developed for use in LMIC settings and translating to a high-income country [40]. Traditionally in clinical research, innovation is scaled from high-resource settings to low-resource settings, resulting in predictable challenges with resource limitations impacting feasibility. Reverse innovation allows researchers to derive insights from LMIC implementation research and innovatively adapt for higher-income settings. Given the importance of trust in promoting vaccine uptake, the current study will amplify the voices of trusted local leaders within historically disadvantaged communities. Emerging research illustrates the popularity of mHealth interventions to extend service capacity and reach, promote healthcare engagement, reduce stigma, and promote vaccine acceptance [41]. The use of digital health products has significantly increased during the COVID-19 pandemic era [42]. Important considerations for the implementation of mHealth in real-world settings include technology literacy, personalization of interventions, and perceptions of health risk and status [43]. We will address these considerations to inform the appropriate selection of implementation strategies tailored for context and implementation phase [44].

#### Care coordination

In addition to information on vaccine effectiveness and access through mHealth strategies, additional support with more intense coordination might be needed for some community members. Care coordination is an evidence-based practice that is widely recognized as a core component of high-quality care [45]. It is also a pillar of the patient-centered medical home (PCMH), which is a model of primary care increasingly adopted by healthcare systems, including FQHCs, to meet the needs of diverse patients and to improve experiences, outcomes, safety, and system efficiency for patients, providers, and staff members [46, 47]. We define care coordination as “the deliberate organization of patient care activities between two or more participants involved in a patient’s care to facilitate the appropriate delivery of health care services” [48–50]. Through personalized health education and care management and collaborative problem-solving, care coordinators will work with patients to develop and achieve a health action plan in collaboration with their primary care physician. The National Committee for Quality Assurance (NCQA) leads the most widely adopted evaluation program for PCMH accreditation, called the NCQA Recognition program [51]. Our FQHC healthcare partner has successfully obtained and maintained NCQA recognition, due, in part, to their care coordination program. This study will expand eligibility for care coordination to target reduction of outstanding preventive services, including but not limited to diabetes and cancer screenings, and influenza vaccination, and offer co-created enhancements to maximize cultural relevance to priority patient communities served by the FQHC.

Taken together, these three implementation strategies — co-creation led by Community Weavers, mHealth, and care coordination — have strong potential to accelerate efforts to sustain COVID-19 vaccination uptake and recommended use of preventive services. Research indicates that social determinants of health, such as low SES, educational level, and access to transportation, along with identifying as members of immigrant, refugee, Latino, and BIPOC communities, reduce one’s likelihood to engage in evidence-based preventive services [52, 53]. As a response to the COVID-19 pandemic, many preventive services were delayed, resulting in more members of underserved communities having overdue preventive service needs [54]. Through a multicomponent health program, COVID-19 vaccination will be coupled with engagement of members of underserved communities in broader preventive services. Engagement in preventive services will be addressed through all components of the program including co-creation of strategies, mHealth outreach, and care coordination. Combining care coordination with culturally relevant, linguistically appropriate, and co-created mHealth health promotion messages offer a powerful approach to both facilitating vaccine equity and preventive services engagement.

### Theory of change and implementation science framework

Our study is guided by a theory of change developed by our team through our National Institutes of Health (NIH)-funded research studies and the practical, robust implementation, and sustainability model (PRISM).

#### Theory of change (ToC)

The multicomponent health program will be informed by an overarching ToC that was developed based on the team’s prior work with two CABs. The goal of the CO-CREATE CAB was to directly inform co-created implementation strategies for a tailored COVID-19 testing program and included 22 members representing community residents, public health researchers, and clinical partners. The goal of the STOP COVID-19 CA CAB was to inform materials and resources needed to support vaccine clinical trial participation and equity initiatives in underserved communities and included 11 community leaders representing ten local grassroots community organizations and two policy partners [23, 24]. ToCs have been broadly used to guide the planning, implementation, and evaluation of public health programs in low-resource settings and provide a comprehensive description of how and why a desired change is expected to happen in a particular context [55, 56]. In these two COVID-19 projects, a rigorous, multistep process was used to create a separate ToC for each CAB [24] which were overlaid to create an overarching, combined ToC. This combined ToC will be confirmed and refined and used to inform our strategy selection and implementation and evaluation planning.

#### Practical, Robust Implementation, and Sustainability Model

The Practical, Robust Implementation, and Sustainability Model (PRISM) will guide the optimization, implementation, and evaluation of our multicomponent health program [57, 58]. PRISM allows for multi-level conceptualization of implementation efforts (context domains) and also provides guidance on how to measure relevant proximal implementation outcomes through integration of the RE-AIM measures (reach, effectiveness, adoption, implementation, maintenance) — one of the most widely used sets of implementation measures [59]. PRISM is ideal for guiding our real-world community-based research project because it accounts for multi-level effects; it builds on several implementation science frameworks and can guide development, implementation, and evaluation. Multiple levels of context will be considered: recipient characteristics (community members and delivery agents — Community Weavers and care coordinators), intervention characteristics (perceived by diverse partners), implementation and sustainability infrastructure within communities and clinics, and the external environment (national guidelines and regulations). These factors will be considered during the co-creation and optimization of the multicomponent health program and assessed dynamically across the lifetime of the study. Several studies show that the reach, adoption, implementation, and sustainment of interventions are related to how well the intervention is integrated into the local context [59–61]. These factors will be assessed in addition to effectiveness, using the reach, effectiveness, adoption, implementation, and maintenance (RE-AIM) framework.

### Aims

The specific aims of the study are as follows:

- Aim 1: Optimize a multicomponent health program to promote COVID-19 vaccine uptake and engagement in preventive healthcare using our established co-creation approach to address multi-level (individual, community, systemic) barriers to vaccine uptake and preventive services engagement.
- Aim 2: Evaluate the implementation, effectiveness, and sustainment of the multicomponent COVID-19 vaccine and preventive services engagement program using a hybrid type 3 effectiveness-implementation sequential multiple assignment randomized trial (SMART) design across immigrant, refugee, Latino, and BIPOC communities in Central, North, and East San Diego.

## Methods

### Design

**Fig. 1:**
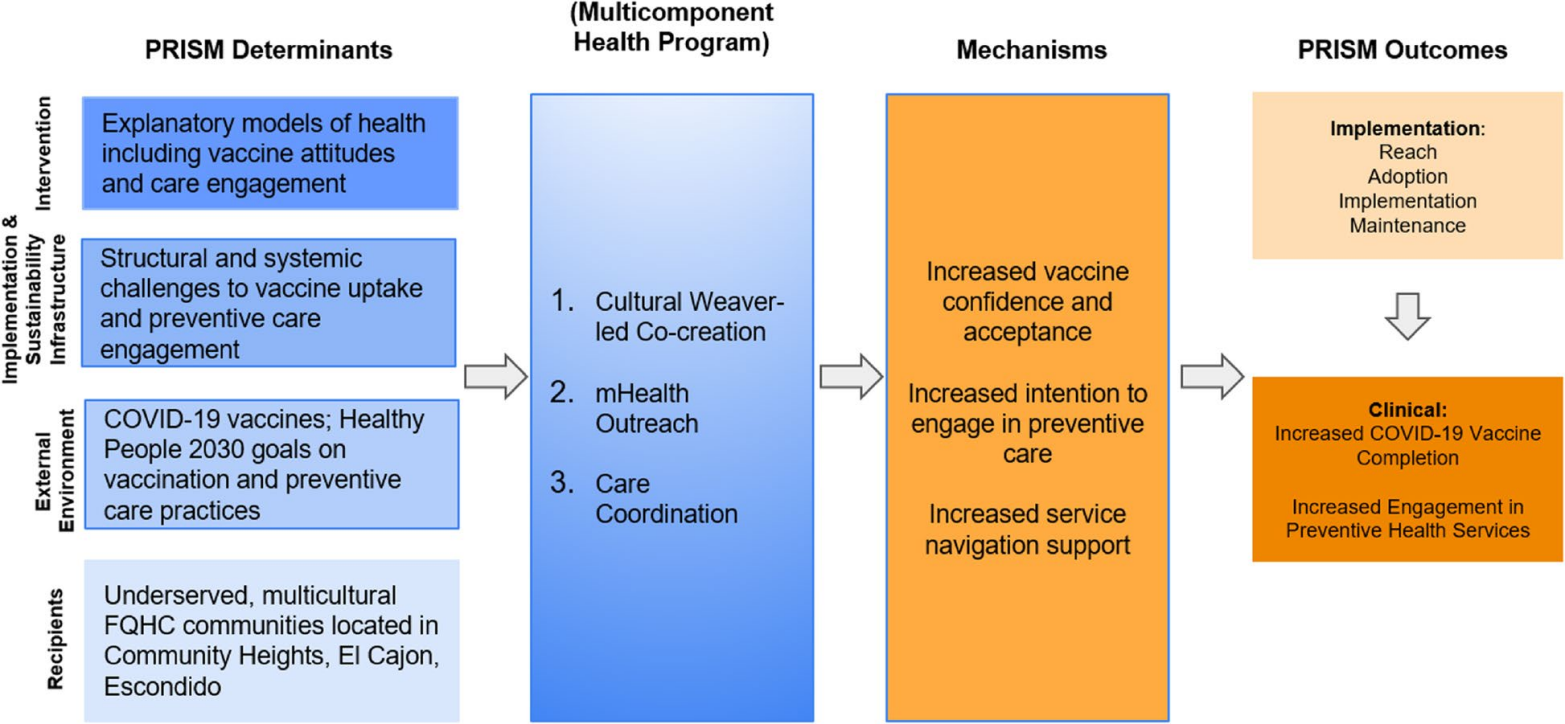
Conceptual model

We will optimize, implement, and test the impact of a multicomponent health program that includes three implementation strategies: co-creation led by Community Weavers, mHealth outreach, and care coordination (described earlier). A participatory approach will be used to engage community members in co-creating and optimizing mHealth outreach and care coordination program components (Aim 1). A hybrid type 3 effectiveness-implementation SMART design will assess the impact of the multicomponent health program on implementation and clinical outcome measures (Aim 2) [62, 63]. See Fig. 1 for the study’s conceptual model.

Through the team’s ongoing community-engaged COVID-19 studies and guided by PRISM, determinants that underlie COVID-19 testing and vaccine disparities in underserved communities (immigrant, refugee, BIPOC, and Latino families) were identified. These determinants include unique explanatory models of health and COVID-19 (intervention PRISM domain), structural and systemic challenges to vaccination and preventive services engagement (implementation and sustainability infrastructure PRISM domain), FDA-approved or authorized COVID-19 vaccines available to all adults (external environment PRISM domain), and the varying needs and characteristics of underserved communities located in our target geographic regions (recipients PRISM domain). It is hypothesized that the selected implementation strategies will exert their impact on implementation and clinical outcomes through vaccine confidence and service navigation support (mechanisms). Implementation outcomes are based on RE-AIM and expected to affect our clinical outcomes (effectiveness).

Clinical outcomes are based on the Office of Disease Prevention and Health Promotion’s Healthy People 2020 objectives [64]. A hybrid type 3 design was selected because there is strong evidence for our clinical interventions (highly effective COVID-19 vaccines and evidence-based preventive services) and effectiveness data for the selected implementation strategies delivered individually. The study’s experimental design follows a SMART randomized structure to introduce the more intensive care coordination component only to those who are not responding to usual care or the lower intensity and less-resource intensive mHealth messaging component allowing for more efficient resource allocation. The Community Weaver-led co-creation component will be included in both mHealth and care coordination since this component is at the heart of the meaningful community engagement and the Community Weaver’s role will be critical for both strategies.

**Fig. 2:**
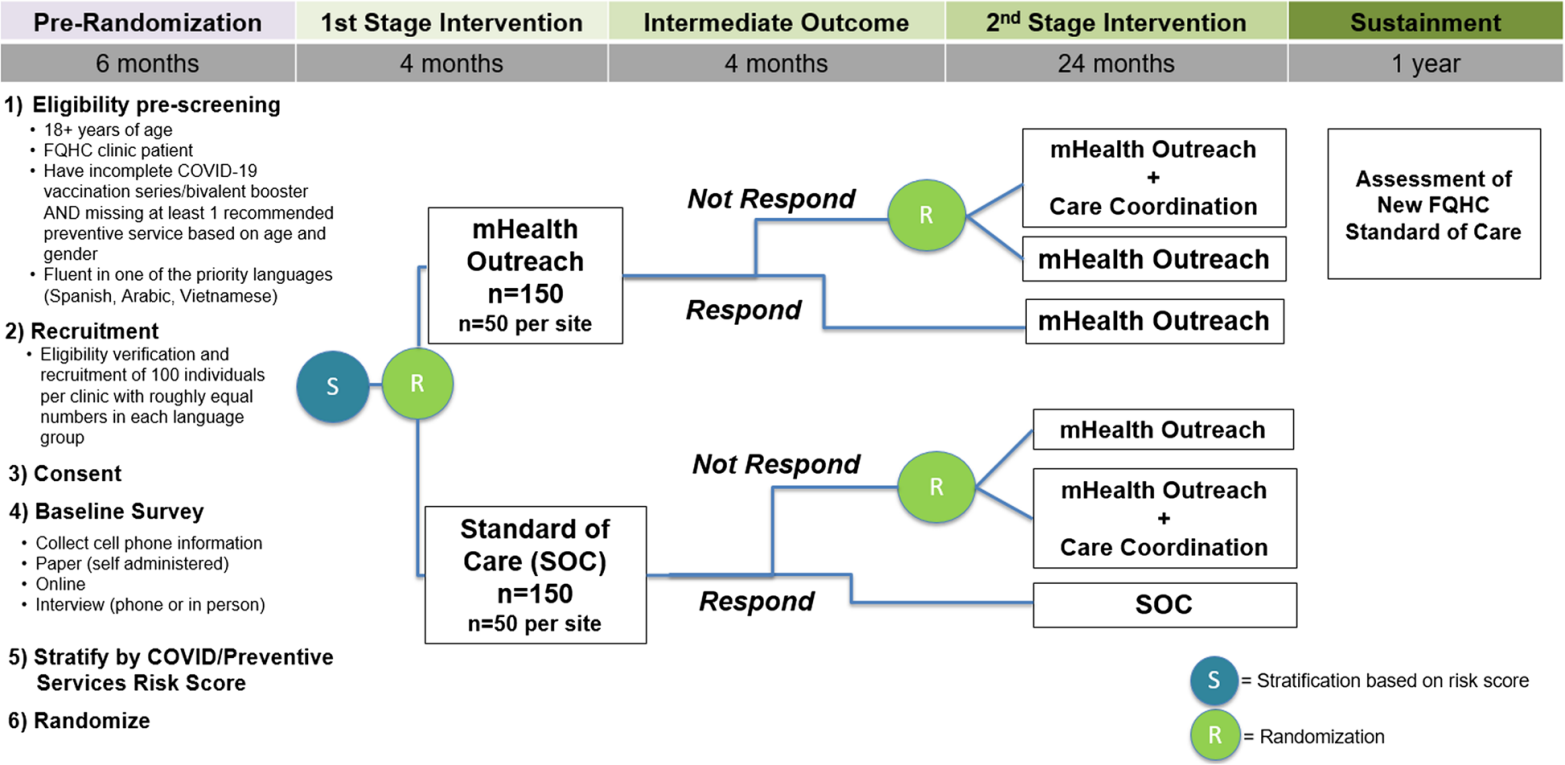
Sequential, multiple assignment randomized trial (SMART) design

The multicomponent strategy is an adaptive approach that utilizes tailored mHealth messaging and care coordination to increase COVID-19 vaccine uptake. The SMART assignment arms are described in Table 2. The trial will span 4 years inclusive of 3 years of active implementation and 1 year of sustainment/maintenance. As outlined in Fig. 2, both randomization and analysis will be conducted at the individual participant level. At enrollment, participants will be stratified by current COVID-19 vaccination status and status of other preventive services vis-à-vis a risk score and then randomized into the first-stage intervention (mHealth outreach or standard of care). Response or lack of response after the completion of the first stage intervention will determine if participants continue in their initial first-stage intervention or re-randomized to increased intensity intervention. Nonresponders in the standard of care arm will be equally re-randomized into either mHealth outreach or care coordination for stage 2. Nonresponders in mHealth outreach will be re-randomized into mHealth or mHealth plus care coordination for stage 2. Nonresponse will be defined as not being up to date on COVID-19 vaccination OR having at least one outstanding preventive service need.

### Setting

This study will be conducted in partnership with the second largest FQHC serving low-income and uninsured San Diegans and has a long-standing relationship with UC San Diego. This study will focus on clinics in Central, North, and East San Diego that serve refugee, immigrant, BIPOC, and Latino populations. San Diego has historically been one of the California counties with the highest number of immigrant and refugee families [65]. This FQHC has been successful in the early phases of COVID-19 vaccine campaigns in many regions but anticipates challenges with sustaining the level of support needed to keep up with guidelines as policy changes supporting COVID-19 vaccine efforts may wane in coming months and years. As shown in Table 1, the three target clinics serve a racially/ethnically diverse patient population whose primary preferred languages are Vietnamese (clinic 1), Arabic (clinic 2), and Spanish (clinic 3). Engagement in preventive services as shown by recommending screenings is lower than recommended in all clinics. In addition, the FQHC has a robust care coordination program in place as part of their PCMH accreditation. Taken together, these factors well position the partnering FQHC to collaboratively deliver a multi-level community-focused intervention to support equitable vaccine uptake and preventive services engagement.

**Table 1:** Patient characteristics by participating Federally Qualified Health Center clinics

	Clinic 1	Clinic 2	Clinic 3
**Patient volume**			
Total no. of patients/site	2573	1751	1528
**Race/ethnicity**			
Asian	44%	68%	5%
Black	9%	2%	5%
White	26%	24%	55%
Latino	22%	7%	44%
**Preferred language**			
Arabic	0.1%	68%	0.4%
Spanish	7%	3%	18%
Vietnamese	32%	No data	0%
**Preventive health services**			
Blood pressure (any time)	74%	93%	89%
HbA1c screening (any time)	57%	56%	71%
**Community-level data by zip code**^**a**^			
COVID-19 positivity (rate per 100,000)	22,589	24,530	23,427
COVID-19 vaccination (rate per 1000)	807	745	803

^a^Source: County of San Diego Health and Human Services Administration from 2022

### Participants

A total of 300 adult patients (100 per clinic), from a sampling frame of approximately 5000 patients, will be recruited to participate in the study. Patient recruitment will occur at each of the three participating FQHC clinics through collaborative efforts between the FQHC’s care coordinators and the Community Weavers. The CABs will be invited to co-develop culturally appropriate recruitment methods to ensure participation from Spanish, Arabic, and Vietnamese-speaking patients connected with the partnering FQHCs. A weighted sampling approach will be considered if patient volume varies considerably between the three clinics during the baseline period. Furthermore, to understand reach of the program, characteristics of those participating in the study will be compared to the population in each of the three geographic regions recruited into the study.

### Procedures

#### Community engagement through CABs and Community Weavers

The multicomponent strategy will be developed and optimized using the team’s established co-creation approach with guidance from the Community Weavers and engagement of the community through CABs. Community Weavers will be hired and supervised by the study’s community partner, the Global ARC, based on their established relationships with ethnically based community organizations. Hiring criteria for Community Weavers include that they are fluent in English and at least one of the three priority languages (Spanish, Arabic, Vietnamese); are engaged with communities living near one of the three partnering FQHC clinics; have experience with community engagement, leading and organizing; and have the capacity to share back knowledge and information with their community as well as share the community’s perspectives and concerns with the CABs.

Community Weavers will have a critical role in developing and refining tailored mHealth messages [66]. They will also be instrumental in facilitating the existing care coordination efforts at the FQHC by focusing on immigrant and refugee communities that can be difficult to engage in care. The Community Weavers will lead three CABs that include five members each inclusive of the three primary languages spoken by community members of our clinics of interest. CAB members will be compensated for their time. In addition to the five community members, we will include the two care coordinators, a mHealth Specialist, and two clinical investigators as nonvoting members in each CAB.

During the first year, each CAB will meet up to four times. These initial meetings will be tasked with co-creating culturally and linguistically appropriate content and frequency for mHealth outreach and identifying topics, strategies, and processes for the care coordination program. mHealth outreach messages will be developed in the native languages of the CABs, and we will use professional translation as needed. To facilitate mHealth message development, Community Weavers will use a storytelling/narrative framework approach inviting residents to join open dialogue and share their journey through the decision-making process towards getting vaccinated, reflecting on the anxieties they navigated, the misinformation they were confronted with, and any structural or technological barriers they had to overcome to enhance the cultural resonance and tailoring of the mHealth messages [67]. In subsequent years, the CABs will continue meeting quarterly to revise and update mHealth content and engage in an Appreciative Inquiry Process to evaluate the implementation of the multicomponent health program and determine when refinements for optimization are needed. The co-creation and optimization of the program components will be guided by the contextual domains of PRISM (i.e., intervention characteristics, recipient characteristics, implementation and sustainability infrastructure, external environment) and will build on the team’s prior work with the community [24, 31] and the CAB members’ lived experience as members of their respective communities.

#### mHealth development and outreach

**Fig. 3:**
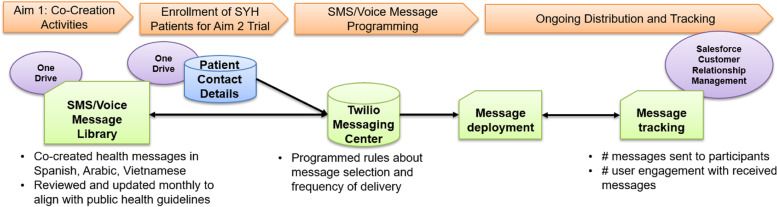
Schematic of the mHealth outreach system

mHealth-related communications will include personalized short messaging service (SMS) and automated phone calls recorded by community leaders in their respective languages aligned with the language preferences of our target clinics. The content of mHealth outreach will include information such as how and where to get vaccinated, myths about COVID-19 and other vaccines, the importance, timing, and scheduling of priority preventive services, and provide reminders for clinical care appointments. The study team includes an expert consultant in health communications to guide message development. mHealth distribution frequency will be determined by each CAB to align with their community’s preferences and needs. New messages will be developed and added to the message library based on updated public health guidance and feedback from participants. The mHealth outreach system will be user tested and refined prior to launch. See Fig. 3 for a schematic of the mHealth outreach system.

A mHealth specialist will lead the development of the mHealth delivery system by consulting with programming experts through Twilio to design and build a fully integrated, Health Insurance Portability and Accountability Act (HIPAA) compliant communications platform to manage the distribution of SMS and voice communications. Twilio will allow customized communications, develop production-ready applications, and run serverless applications on one application programming interface-powered platform. A centralized study database connected with the mHealth delivery system will track the number and timing of text and voice messages sent to individual participants. User analytics will be captured such as individual reach, responsiveness, and engagement. Example messages may include the following: “Proteja a sus seres queridos con vacunarse contra el COVID-19”/ “Protect your loved ones by getting the COVID-19 vaccine” and “Recuerde pedir su vacuna contra la Influenza”/ “Remember to ask for your flu shot.”

#### Care coordination

The partnering FQHC has an established process to provide care coordination (see standard of care in Table 2). The partnering FQHC will identify study-specific care coordinators who are members of the three study communities. Care coordination for the intervention will be tailored to the needs and cultural preferences of the respective patient community through input from our CABs. The content of care coordination will include engagement of patients in preventive services, developing a patient health action plan, support with adhering to the health action plan, scheduling preventive and care management appointments, communicating with the patient’s care team to ensure continuity of care, and COVID-19 vaccination.

**Table 2:** Summary of the Sequential, Multiple Assignment, Randomized Trial (SMART) design

Standard of Care	Multi-component health program
Aligned with patient-centered medical home components and quality-of-care measures	**Community Weaver led Co-Creation** Overarching strategy
**Care coordination**	**mHealth Outreach**
• Routine immunization efforts • Annual physical exam • Eligibility: defined medical conditions (e.g., uncontrolled diabetes, hypertension), high health services utilization (e.g., > 5 medications), social determinants of health and referred by care team	Community outreach led by Community Weavers via SMS and voice messages about COVID-19 vaccines (importance, reminders, how to access) and preventive services engagement (importance, reminders, how to access) in languages spoken by patients (i.e., Spanish, Arabic, Vietnamese)
	**Care Coordination**
	Inclusive of standard of care coordination with added focus on completing outstanding preventive services (e.g., HbA1c, blood pressure screenings, mammograms, colorectal cancer screening)

### Measures

Data will be collected using multiple sources to represent diverse perspectives using a QUANT + QUAL concurrent mixed-method design approach. Data sources, type, source, and timing of collection as well as key implementation and effectiveness outcomes are summarized in Tables 3 and 4. Surveys and interviews with participants will be conducted in the participant’s preferred language. Participant surveys will be drafted with input from the CABs, pilot tested prior to implementation, and use electronic or paper formats based on participant preferences. Interviews will be led by Community Weavers using a semi-structured interview guide reflecting PRISM domains adapted from prior studies [58, 72] and vetted by the CABs. Interviews will be recorded and translated to English. Study data for mHealth message delivery, care coordination, and Community Weavers will be stored in Research Electronic Data Capture (REDCap). Periodic reflections will be collected as weekly diary entries from members of the research team. Partner engagement will be evaluated using a concurrent mixed-methods approach developed through the team’s NIH-funded COVID-19 projects [68]. A partner engagement survey (Goodman et al.) will be distributed following each CAB meeting to assess how well and how often eight engagement principles were perceived during the meeting [69]. Ethnographic documentation of the CAB process will be conducted by trained research staff using a structured documentation form identifying various aspects of engagement (e.g., topics covered, time spent contributing) [25].

**Table 3:** Main data sources, data types, origin, and the domains of the practical, robust implementation and sustainability model (PRISM)

Data source name	Type	Origin	PRISM domains
mHealth message delivery study database (QUANT + QUAL)	#, timing, type, content of message delivery for each language groups	mHealth specialist	Implementation Maintenance
Care coordination study database (QUANT + QUAL)	#, timing, type of care coordination activities	Care coordinators	Implementation Maintenance
Community Weaver study database (QUANT + QUAL)	#, timing, type of interactions with communities	Community Weavers	Implementation Maintenance
Periodic reflections (QUAL)	Key lessons learned, facilitators, challenges of implementation of and adaptations to program components	mHealth specialist Community Weavers Care coordinators Research team	Intervention characteristics Implementation & sustainability infrastructure External environment
Participant user engagement check-in (QUANT)	Acceptability, appropriateness, utility, and value of text/voice messages and care coordination	All participants in mHealth or mHealth & care coordination arm	Reach Implementation Intervention characteristics
Participant evaluation surveys (QUANT)	Satisfaction, engagement, and impact of the multicomponent health program	All participants in mHealth or mHealth and care coordination arm	Implementation Effectiveness Maintenance
Participant interview (QUANT + QUAL)	Acceptability, appropriateness, utility, and value of text/voice messages and care coordination	Subset of participants (*n* = 60, 20 from each clinic)	Implementation Effectiveness Maintenance
Participant vaccine and preventive service status & health characteristics (QUANT)	EHR data on study primary and secondary outcomes, patient demographic, other health characteristics	Care coordinators	Reach Recipient characteristics
Delivery agent survey (QUANT)	Demographic and professional characteristics of Community Weavers and care coordinators	Community Weavers Care coordinators	Recipient characteristics Adoption

*QUANT*, quantitative; *QUAL*, qualitative

**Table 4:** Outcome measures summarizing the implementation and clinical effectiveness outcomes

Implementation outcomes	Reach	The absolute number, proportion, and representativeness of participants who are compared to all eligible patients on key characteristics. Reasons for nonparticipation
	Adoption	Demographic and professional characteristics of Community Weavers and care coordinators
	Implementation	#, timing, type, content of delivery of mHealth messages, care coordination, and Community Weaver activities
	Maintenance	Ongoing delivery of mHealth messages, care coordination, and Community Weaver activities during the sustainment phase. Ongoing impact on vaccine acceptance and confidence, COVID-19 vaccine uptake, and preventive services engagement
Effectiveness (clinical) outcomes	Vaccine confidence	Adapted vaccine confidence survey
	Intention to engage in preventive services	Measured as scheduled appointments for due or overdue preventive services
	COVID-19 vaccine uptake	Up-to-date vaccine record defined as completed vaccine series and required boosters as recommended by Centers for Disease Control guidelines for COVID-19 vaccination at the time of implementation to be confirmed through electronic health records
	Preventive services engagement	Up-to-date age- and gender-specific preventive services (e.g., mammograms, adult immunizations, blood pressure screenings, A1c screenings, colorectal cancer screenings). Standards will be defined for each participant based on their demographic and health characteristics

### Power calculations

Primary effectiveness outcomes will be COVID-19 vaccine (initial and bivalent booster) completion at a sustained rate of 70% (consistent with Healthy People 2020 influenza vaccination goal for adults aged 18 + years) [70]. Secondary effectiveness outcomes will be an increase in preventive service engagement defined as completing a physical exam and at least one recommended preventive care screening (e.g., mammogram, A1c screening, colorectal cancer screening) annually and increases in vaccine confidence. Data on other recommended adult immunizations (e.g., influenza, shingles) will be extracted from patient electronic health records (EHRs). Primary implementation outcomes will be reach, adoption, implementation, and maintenance up to 2-year post-implementation. Target sample size was determined using methods described by Kidwell et al. [71] for a SMART design with a binary outcome. A power analysis was conducted to inform our targeted sample size based on the following assumptions: (1) probability of success for adaptive health interventions of 0.70; (2) estimated response rate for standard of care of 0.45 (current vaccination rate among target population); and (3) equal re-randomization after first-stage intervention for nonresponders. Allowing for a 5% type-1 error and 80% power, a sample size of at least 252 is needed to evaluate an effect size of 0.25. Assuming a 15% loss to follow-up during the 2-year intervention period, our target enrollment of 300 participants will ensure sufficient sample size for analysis.

### Analytic approach

Quantitative data from surveys, study databases, and EHRs will be summarized using simple descriptive statistics along with data visualization methods to understand patterns and characteristics of responders and nonresponders and to guide adaptive implementation. Parametric and nonparametric tests will be employed to compare the survey responses between the arms of the trial variables and clinic locations. Factors will initially be assessed at the univariate level and then used to build multivariable models to assess independent associations of multiple factors on vaccination uptake, including community level factors such as prevalence of COVID-19 vaccination, history of COVID-19 infection, and primary community language group. Multinomial logistic regression models will explore the relation between standard of care, mHealth outreach, and mHealth plus care coordination and COVID-19 vaccine uptake, while controlling for a variety of demographic and socioeconomic variables and comorbidities, as well as FQHC clinic characteristics. Additionally, the association between adaptive interventions and the secondary clinical composite outcome, preventive services engagement, including age and gender-specific services will be evaluated. Descriptive analysis will be used to characterize within subgroup differences. Mixed-effects logistic regression models will be used to identify factors associated with COVID-19 vaccine uptake and vaccine confidence using EHR and demographic data collected with participant evaluation surveys.

Qualitative data will be culled from interviews, periodic reflections, and study databases. All qualitative data will be coded using a rapid qualitative data analytic approach guided by the domains of PRISM, informed by prior work of the lead and senior authors across diverse contexts [58, 72, 73]. This approach involves developing a templated matrix of summary responses from interviews, followed by coding by a qualitative analytic team based on pre-defined themes while allowing for new sub-themes to emerge [74]. Group coding for consensus building, team-based review of codes, and double coding to verify acceptable inter-rater reliability will occur throughout the coding process. Community Weavers and clinic partners will be invited to review the coded data to support sense-making and develop action items based on the data.

A matrix approach will be used to triangulate data from quantitative and qualitative sources using the key domains of PRISM. A joint display analysis will be produced to support the integration of data sources [75, 76]. Unique and common themes across language groups will be identified and described.

## Discussion

This implementation study capitalizes on the unique and complementary methods from community engagement, implementation science, health equity, health communication, infectious disease, and public health to co-create a multicomponent strategy to promote sustained uptake of effective public health approaches of COVID-19 vaccination and preventive services engagement for underserved communities in San Diego. This proposal extends the team’s current work [23, 24, 77] using a generalizable co-creation approach to focus on scaling and sustaining COVID-19 and broader health equity for immigrant, refugee, Latino, and BIPOC communities.

This study will contribute to the limited evidence base in implementation science on public health scaling and sustainment strategies by conducting a hybrid type 3 effectiveness-implementation study on a multicomponent strategy co-created with community leaders, residents, and frontline providers. Limited research exists on sustainment strategies so the explicit focus on scaling and sustaining will contribute to the field of implementation science by adding evidence to health equity-promoting sustainment strategies. This study will include a multi-level mixed-methods data collection approach at multiple time points guided by an implementation science model and is designed to assess sustained impact of our multicomponent program with a 12-month follow-up period. An adaptive implementation approach operationalized in a SMART design will be used to allow deployment of various components of our multicomponent program over time depending on the response to prior components. This approach will facilitate understanding the impact of various intervention components and delivering more intensive support those who benefit from it most. Finally, while the mHealth messaging will target COVID-19 vaccines and boosters, generalizable knowledge will be garnered that may support development of messages for other immunizations if this intervention is to be expanded and scaled.

Balanced with these innovative features are potential challenges and limitations. Based on the team’s ongoing COVID-19 work, a primary challenge will be balancing reach with resource constraints. The underserved communities who reside in the partnering FQHC clinic regions are diverse in their cultures, languages, explanatory models of health and COVID-19, and healthcare needs. It will not be possible to engage all immigrant, refugee, and BIPOC communities through our co-created health program. However, the team will rely on the decades of community engaged participatory action research led by the Global ARC to successfully reach a diverse range of immigrant and refugee communities in ways that are meaningful, sustainable and resource appropriate, and leverage the quality care and clinical research supported by the partnering FQHC to reduce health disparities in their patient population.

This study has great potential to contribute to implementation science through the operationalization of a relatively infrequently operationalized SMART design, the use of longitudinal, multi-level, and mixed-methods data collection informed by an implementation science framework, and the use of innovative implementation strategies of co-creation through CABs led by Community Weavers and care coordination. It is expected that the study will have meaningful population health impact by increasing COVID-19 vaccine and preventive services uptake for key historically underserved populations. Furthermore, findings of this study have potential to uniquely unpack the phenomenon of health and vaccine misinformation that was exacerbated through the COVID-19 pandemic [78]. In closing, this study has potential implications beyond a narrow focus on COVID-19 disparities reduction to influence broader public health implementation efforts for underserved communities.

## Data Availability

Not applicable.

## Abbreviations

BIPOC: Black, Indigenous, and People of Color
CDC: Centers for Disease Control and Prevention
EHR: Electronic health records
FDA: Federal Drug Administration
FQHC: Federally Qualified Health Center
Global ARC: Global Action Research Center
HIPAA: Health Insurance Portability and Accountability Act
LMIC: Low- or middle-income country
NCQA: National Committee for Quality Assurance
NIH: National Institutes of Health
PCMH: Patient-centered medical home
PRISM: Practical, robust implementation and sustainment model
QUANT: Quantitative
QUAL: Qualitative
RE-AIM: Reach, effectiveness, adoption, implementation, and maintenance
REDCap: Research Electronic Data Capture
SES: Socioeconomic status
SMART: Sequential multiple assignment randomized trial
SMS: Short message/messaging service
ToC: Theory of change
US: United States
